# Editorial: Probiotics and constipation

**DOI:** 10.3389/fnut.2022.1114149

**Published:** 2023-01-04

**Authors:** Jiajia Song, Xin Zhao, Kun-young Park, Huayi Suo

**Affiliations:** ^1^College of Food Science, Southwest University, Chongqing, China; ^2^Chongqing Collaborative Innovation Center for Functional Food, Chongqing University of Education, Chongqing, China; ^3^Department of Food Science and Biotechnology, Cha University, Seongnam-si, Republic of Korea

**Keywords:** probiotics, constipation, gut microbiota, mechanism, synbiotics

Constipation is a common disorder of gastrointestinal motility, which usually causes infrequent stools, and difficulty in the passage of stools. Many aspects, including genetic predisposition, daily diet and behavior, socioeconomic status, and other biological and clinical factors are considered to be associated with the pathogenesis of constipation (1). Constipation affects individuals of all ages, especially the elderly, and may result in more serious complications such as fecal incontinence, hemorrhoids, and anal fissure (2, 3). Thus, developing effective strategies for the management of constipation is essential. Dysbiosis of intestinal microbiota has been associated with constipation, and the restoration of gut microbiota homeostasis is proposed as a promising strategy for the treatment of constipation (4, 5). Currently, animal and clinical studies have shown that several probiotics (live microorganisms) can attenuate constipation by regulating the gut microbiota (6–8). However, the identification, effectiveness evaluation, and underlying mechanism of novel probiotics relieving constipation are still necessary because of the strain specificity of probiotic effects.

This Research Topic mainly focuses on the attenuation effect and potential mechanism of new probiotics on constipation. To date, five papers, including four research articles and one review article have been collected in this Research Topic. *Bacillus coagulans* BC01, *Bifidobacterium lactis* TY-S01, and *Lactobacillus plantarum* KFY02 increased the fecal moisture and gastrointestinal transit rate in mice (Zhou et al., Tang et al. and Yi et al.). Furthermore, the authors found that these strains can promote the homeostasis of gut microbiota in the constipated mice (Zhou et al., Tang et al., and Yi et al.). *Bifidobacterium lactis* TY-S01 and *Lactobacillus plantarum* KFY02 increase the community richness and bacterial diversity. More importantly, the structure and composition of intestinal flora in the constipated mice are markedly changed by these three strains, and the change in the abundance of special species depends on the strain specificity of probiotics. The mechanisms underlying these effects on constipation are summarized as follows.

## 1. The production of short-chain fatty acids (SCFAs)

*Bifidobacterium lactis* TY-S01 increases the content of SCFAs, such as isobutyrate acid, butyrate acid, acetic acid, valeric acid, and propionic acid, in the feces of the constipated mice (Tang et al.). These SCFAs may inhibit the growth of pathogenic bacteria by reducing the pH in the small intestine, and improve gut motility by increasing colonic smooth muscle contraction, therefore contributing to the amelioration of constipation symptoms (Araújo and Botelho).

## 2. The maintenance of gut barrier integrity

The imbalance of intestinal bacteria can cause the release of inflammatory cytokines, thereby damaging the integrity of gut barrier (Yi et al.). The destruction of gut barrier may result in the further invasion of pathogenic bacteria and their toxins, which aggravates intestinal inflammation. The maintenance of gut barrier integrity can inhibit the adherence of pathogens bacteria and their metabolites and facilitate stool passage (Araújo and Botelho). Zhou et al. reported that the nuclear factor (NF)-κB signaling-mediated inflammation in the small intestine of mice with constipation is inhibited by *Bacillus coagulans* BC01. *Bifidobacterium lactis* TY-S01 decreases the mRNA expression of colonic inflammatory cytokines in the constipated mice (Tang et al.). Furthermore, the expression levels of gut barrier-associated key genes, including transient receptor potential vanilloid-1, mucin 2, stem cell factor, claudin-1, occludin c-kit, and glial cell line-derived neurotrophic factor are significantly regulated by *Bifidobacterium lactis* TY-S01 and *Lactobacillus plantarum* KFY02 (Tang et al. and Yi et al.), indicating that these probiotics have the potential ability to restore the gut barrier.

## 3. The secretion of intestinal hormones

The content of motilin, 5-hydroxytryptamine, and substance P in the constipated mice is increased by *Bifidobacterium lactis* TY-S01, and the secretion of these intestinal hormones has a strong correlation with particular species of gut microbiota (Tang et al.). These intestinal hormones can regulate intestinal peristalsis and improve constipation symptoms (Araújo and Botelho and Yi et al.). Besides, Zhou et al. reported that *Bacillus coagulans* BC01 reduce the production of vasoactive intestinal peptide and somatostatin in the constipated mice, contributing to the slowing down of intestinal transit time and the relief of constipation.

Apart from the regulation of gut microbiota in the constipated mice by probiotics, Zhang et al. examined the regulatory effect of synbiotic consisting of *Bifidobacterium lactis* BL-99 and fructooligosaccharide (FOS) on the intestinal flora from constipated individuals *in vitro*. They found that this synbiotic enhances the level of acetic acid, but also changes the structure of intestinal flora, and increases the abundance of beneficial bacteria. However, human and animal studies evaluating the improved effect of synbiotics on constipation are scarce (Araújo and Botelho).

In summary, although the above-mentioned probiotics show a good efficiency in improving the constipation of mice, randomized clinical trials are needed to further confirm their anti-constipation effect. The molecular mechanisms underlying probiotics effect on constipation, including the identification of key metabolites and their mode of action need to be further investigated. In addition, it is necessary to further strengthen the *in vivo* studies and mechanism of synbiotics on constipation.

## Author contributions

JS wrote the whole manuscript. XZ, K-yP, and HS provided some valuable comments. All authors contributed to the article and approved the submitted version.
